# Impaired brain activity in patients with persistent atrial fibrillation assessed by near-infrared spectroscopy and its changes after catheter ablation

**DOI:** 10.1038/s41598-022-12097-5

**Published:** 2022-05-12

**Authors:** Akiomi Yoshihisa, Soichi Kono, Takashi Kaneshiro, Yasuhiro Ichijo, Tomofumi Misaka, Shinya Yamada, Masayoshi Oikawa, Itaru Miura, Hirooki Yabe, Yasuchika Takeishi

**Affiliations:** 1 Department of Cardiovascular Medicine, Fukushima Medical University, 1 Hikarigaoka, Fukushima, 960-1295 Japan; 2 Department of Clinical Laboratory Sciences, Fukushima Medical University School of Health Science, Fukushima, Japan; 3 Department of Neuropsychiatry, Fukushima Medical University, Fukushima, Japan

**Keywords:** Cardiology, Cardiovascular biology, Interventional cardiology

## Abstract

Although the prevalence of cognitive impairment and depression is higher in patients with atrial fibrillation (AF) than in the general population, the mechanism has not been fully examined and impact of catheter ablation (CA) of AF also remains unclear. Recently, the development of near-infrared spectroscopy (NIRS) has enabled noninvasive measurements of regional cerebral blood volume and brain activity, in terms of cerebral oxyhemoglobin in the cerebral cortex. We assessed brain activities by NIRS, depressive symptoms by the Center for Epidemiologic Studies Depression Scale (CES-D) and cognitive function by Mini-Mental State Examination (MMSE). We then compared the results between AF patients (paroxysmal AF n = 18 and persistent AF n = 14) and control subjects (n = 29). Next, we also followed up persistent AF patients who kept sinus rhythm at 3 months after CA (n = 8) and measured their brain activities using NIRS, CES-D and MMSE after CA to investigate the associations of changes in brain activities with changes in both CES-D and MMSE. Our results showed that (1) frontal and temporal brain activities were lower in patients with persistent AF than both in control subjects and paroxysmal AF patients (P < 0.01), (2) frontal and temporal brain activities were improved in more than half of the persistent AF patients who kept sinus rhythm at 3 months after CA, especially in those who presented impaired brain activity before CA, and (3) improvement of frontal brain activity was associated with improvement of CES-D (R =  − 0.793, P = 0.019), whereas improvement of temporal brain activity was associated with improvement of MMSE (R = 0.749, P = 0.033). NIRS measurement showed reduced frontal and temporal brain activities in the persistent AF patients, CA improved frontal and temporal brain activities in some of these patients, and associated with improvement of depressive state and/or improvement of cognitive function.

## Introduction

The global burden of atrial fibrillation (AF) is currently on the rise, owing to aging of the society^1,2^. Dementia, mainly manifested as cognitive impairment, is being more common in the elderly population in a similar fashion to AF. Recently, many studies have shown that AF may be a risk factor for cognitive impairment independent of a stroke, suggesting that AF itself has additional adverse effects on the cognitive function^3,4^. In addition, it has recently been reported that catheter ablation (CA) of AF improves the cognitive function assessed by a brief cognitive screening tool (i.e. The Montreal Cognitive Assessment)^5^. On the other hand, Medi et al.^6^ reported that post-procedural neurocognitive dysfunction occurred in 13% to 20% of 90 AF patients after CA. In addition, AF patients have higher rates of depression than healthy subjects^7^. Depression to be more severe in patients with persistent AF than in those with paroxysmal AF^8^. Persistent AF patients were more likely to report more severe depressive state than paroxysmal AF patients^8^. Previous studies have found associations between depression and both increased symptom burden and increased mortality in AF patients, and between depressive symptoms and increased risk of AF recurrence after cardioversion^9^ and after CA^10^. Depression may be associated with dysregulation in the autonomic nervous system, particularly excessive sympathetic activity. Emotional stress has previously been reported to be associated with increased risk of arrhythmias^11^.

Although AF may increase the risk of cognitive impairment or depressive state through silent brain infarction^12^, brain hypoperfusion due to a reduced cardiac output^13^ or inflammation and platelet dysfunction^14^, the mechanism of cognitive impairment or depressive state caused by AF has not been fully examined, and direct imaging has not been performed. In addition, (1) cognitive improvement by CA is controversial, (2) changes in depressive state by CA have not been reported, and (3) direct imaging has not been performed to determine changes in brain perfusion.

Recently, the development of near-infrared spectroscopy (NIRS) has enabled noninvasive and bedside measurements of regional cerebral blood volume in terms of relative concentrations of oxyhemoglobin (oxy-Hb) and deoxyhemoglobin (deoxy-Hb), with a high time resolution. The concentrations of oxy-Hb and deoxy-Hb are assumed to reflect the regional cerebral blood volume^15–17^. In addition, oxy-Hb increases and deoxy-Hb decreases detected by NIRS have been shown to reflect cortical activation by simultaneous measurements with other methodologies, which demonstrate cerebral perfusion and is used as functional brain monitoring^15^. A positive correlation has been observed between oxy-Hb concentration measured by NIRS and blood-oxygen-level-dependent signal measured by functional magnetic resonance imaging (MRI)^18,19^. Furthermore, NIRS has recently been used to investigate the neurocognitive processes associated with neurological (e.g. Alzheimer’s disease, traumatic brain injury) and psychiatric disorders (e.g. depression, anxiety disorders)^20^. Compared to positron emission tomography, single photon emission computed tomography, and functional MRI, NIRS has the advantages of requiring minimal equipment and being easy to use.

In the present study, we aimed to (1) evaluate and compare frontal and temporal brain activities using NIRS in AF patients and control subjects, (2) evaluate changes in brain activities before and after CA in patients with persistent AF who kept sinus rhythm (SR) at 3 months after CA, and (3) determine the associations of changes in brain activity with changes in cognitive function and depressive symptoms.

## Results

The comparisons of clinical features and psychological testing between the control subjects and AF patients are shown in Table 1. We found no significant difference in age, sex, prevalence of co-morbidity, left ventricular ejection fraction, B-type natriuretic peptide, hemoglobin, estimated glomerular filtration rate, percutaneous oxygen saturation, the Center for Epidemiologic Studies Depression Scale (CES-D) was used to evaluate depressive symptoms^21^, or the Mini-Mental State Examination (MMSE) was used to evaluate cognitive function^22^ between the two groups.

**Table 1:** Comparisons of clinical features between the control subjects and atrial fibrillation patients.

	Control subjects (*n* = 29)	Atrial fibrillation patients (*n* = 32)	*P*-value
Type of atrial fibrillation	–	Paroxysmal 18 (56.2) Persistent 14 (43.8)	–
**Demographic data**			
Age (years)	68.9 ± 10.0	68.5 ± 12.3	0.367
Male sex (*n*, %)	22 (75.9)	21 (65.6)	0.414
**Co-morbidity**			
Hypertension (*n*, %)	21 (72.4)	18 (56.3)	0.286
Diabetes (*n*, %)	9 (31.0)	9 (28.1)	1.000
Dyslipidemia (*n*, %)	19 (65.5)	16 (50.0)	0.301
**Laboratory data**			
Left ventricular ejection fraction (%)	60.3 ± 11.5	58.4 ± 11.0	0.523
B-type natriuretic peptide (pg/ml)^a^	46.5 (11.6–96.7)	62.7 (21.7–156.7)	0.322
Hemoglobin (g/dl)	13.0 ± 1.6	13.7 ± 2.2	0.157
eGFR (ml/min/1.73 m^2^)	57.5 ± 13.3	59.1 ± 17.8	0.699
SpO_2_	96.9 ± 1.4	96.8 ± 1.5	0.809
**Psychological testing**			
CES-D	11.0 ± 9.5	11.9 ± 6.3	0.255
MMSE	27.8 ± 2.2	26.9 ± 2.3	0.336

eGFR, estimated glomerular filtration rate; SpO_2,_ percutaneous oxygen saturation; CES-D, the Center for Epidemiologic Studies Depression Scale; MMSE, the Mini-Mental State Examination.

^a^Data are presented as median (interquartile range).

**Figure 1:**
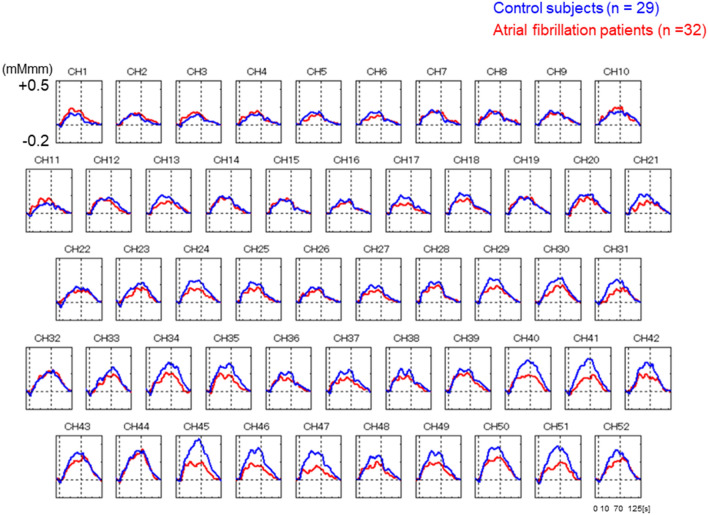
Comparison of grand average of waveform changes in oxyhemoglobin concentrations during verbal fluency task in the atrial fibrillation patients (red, n = 32) and control subjects (blue, n = 29). The horizontal axis represents time, and the vertical axis represents changes in mean oxyhemoglobin concentrations (mMmm) during the verbal fluency task.

**Figure 2:**
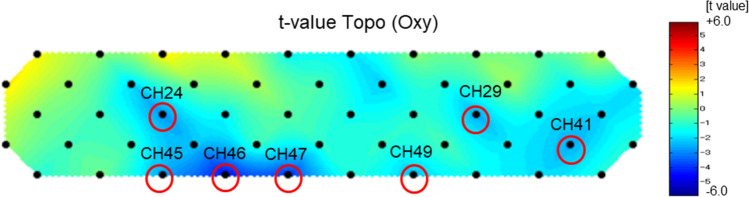
Topographic map of the differences in mean oxy-Hb concentration changes between the atrial fibrillation (AF) patients (n = 32) and control subjects (n = 29). The mean oxy-Hb concentrations in the right temporal lobe (channels 24 and 45), left temporal lobe (channels 29 and 41) and frontal region (channels 45–47 and 49) were significantly lower in the AF patients than in the control subjects (red circle, P < 0.05).

Figure 1 compares grand average of waveform changes in oxyhemoglobin concentrations during verbal fluency task (VFT) in the atrial fibrillation patients (red, n = 32) and control subjects (blue, n = 29). The horizontal axis represents time, and the vertical axis represents changes in mean oxy-Hb concentrations (mMmm) during VFT. Figure 2 shows a topographic map of the differences in mean oxy-Hb concentrations during VFT between the AF patients and control subjects. The mean oxy-Hb concentrations changes during VFT in the right temporal lobe (channels 24 and 45), left temporal lobe (channels 29 and 41) and frontal region (channels 45–47 and 49) were significantly lower in the AF patients than in the control subjects (P < 0.05).

**Figure 3:**
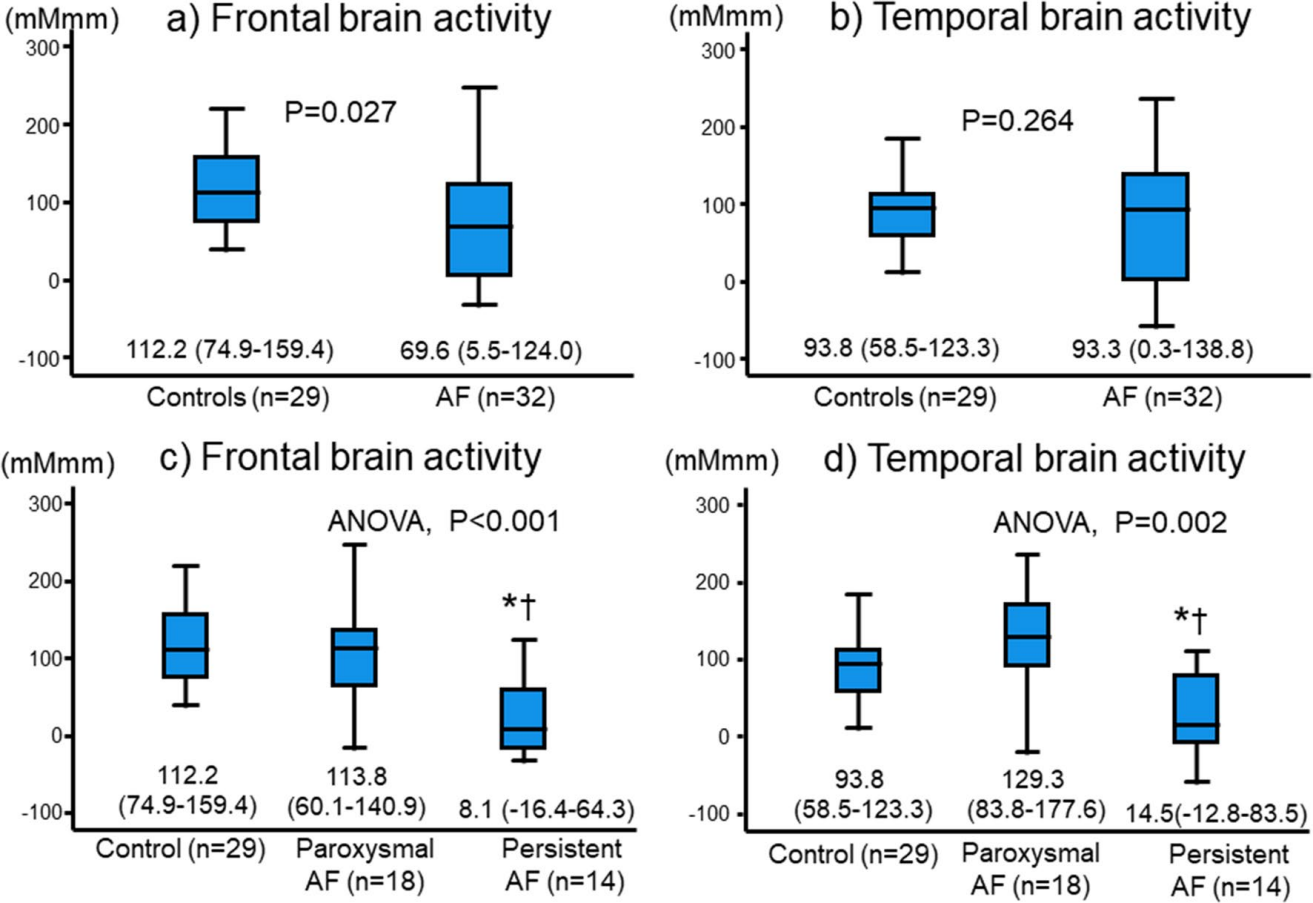
Comparisons of frontal brain activity (integral values of mean oxy-Hb concentrations in the frontal region) and temporal brain activity (integral values of mean oxy-Hb concentrations in the temporal lobes) between the atrial fibrillation (AF) patients and control subjects. Although frontal brain activity was significantly lower in the AF patients than in the control subjects (**a**), temporal brain activity did not differ between the groups (**b**). However, both frontal and temporal brain activities were lower in the persistent AF patients than in the control subjects and paroxysmal AF patients (**c**,**d**). Data is presented as median and interquartile range (error bar).

Next, we compared frontal and temporal brain activities (integral values of mean oxy-Hb concentrations in the frontal and temporal lobes) between the groups, and the results are presented in Fig. 3. Although frontal brain activity was significantly lower in the AF patients than in the control subjects (Fig. 3a), temporal brain activity did not differ between the groups (Fig. 3b). In the multiple regression analysis to determine brain activity confounding factors (Table 2), AF was independently associated with frontal brain activity (β = − 0.356, p = 0.004), but not with temporal brain activity. On the other hand, both frontal and temporal brain activities were lower in the persistent AF patients than in the control subjects and paroxysmal AF patients (Fig. 3c,d). Thus, frontal and temporal brain activities were impaired in the persistent AF patients, rather in the paroxysmal AF, which might have led to a depressive state and cognitive impairment in these patients.

**Table 2:** Multiple regression analysis to determine brain activity confounding factors.

Frontal brain activity	Univariate		Multivariate	
Factors	β coefficient	p value	β coefficient	p value
Age	− 0.293	0.022	− 0.370	0.002
Male gender	0.115	0.376		
Hypertension	0.149	0.259		
Diabetes	− 0.150	0.248		
Dyslipidemia	− 0.050	0.702		
Atrial fibrillation	− 0.282	0.027	− 0.356	0.004
Left ventricular ejection fraction	0.089	0.497		
B-type natriuretic peptide	− 0.215	0.100		
Hemoglobin	0.214	0.098		
eGFR	0.176	0.175		
SpO_2_	0.281	0.030	0.257	0.027

Temporal brain activity	Univariate		Multivariate	
Factors	β coefficient	p value	β coefficient	p value
Age	− 0.227	0.079		
Male gender	0.177	0.173		
Hypertension	0.200	0.122		
Diabetes	− 0.122	0.348		
Dyslipidemia	0.060	0.645		
Atrial fibrillation	− 0.145	0.264		
Left ventricular ejection fraction	0.106	0.418		
B-type natriuretic peptide	− 0.171	0.192		
Hemoglobin	0.226	0.080		
eGFR	0.103	0.429		
SpO_2_	0.240	0.064		

eGFR, estimated glomerular filtration rate; SpO_2,_ percutaneous oxygen saturation.

**Figure 4:**
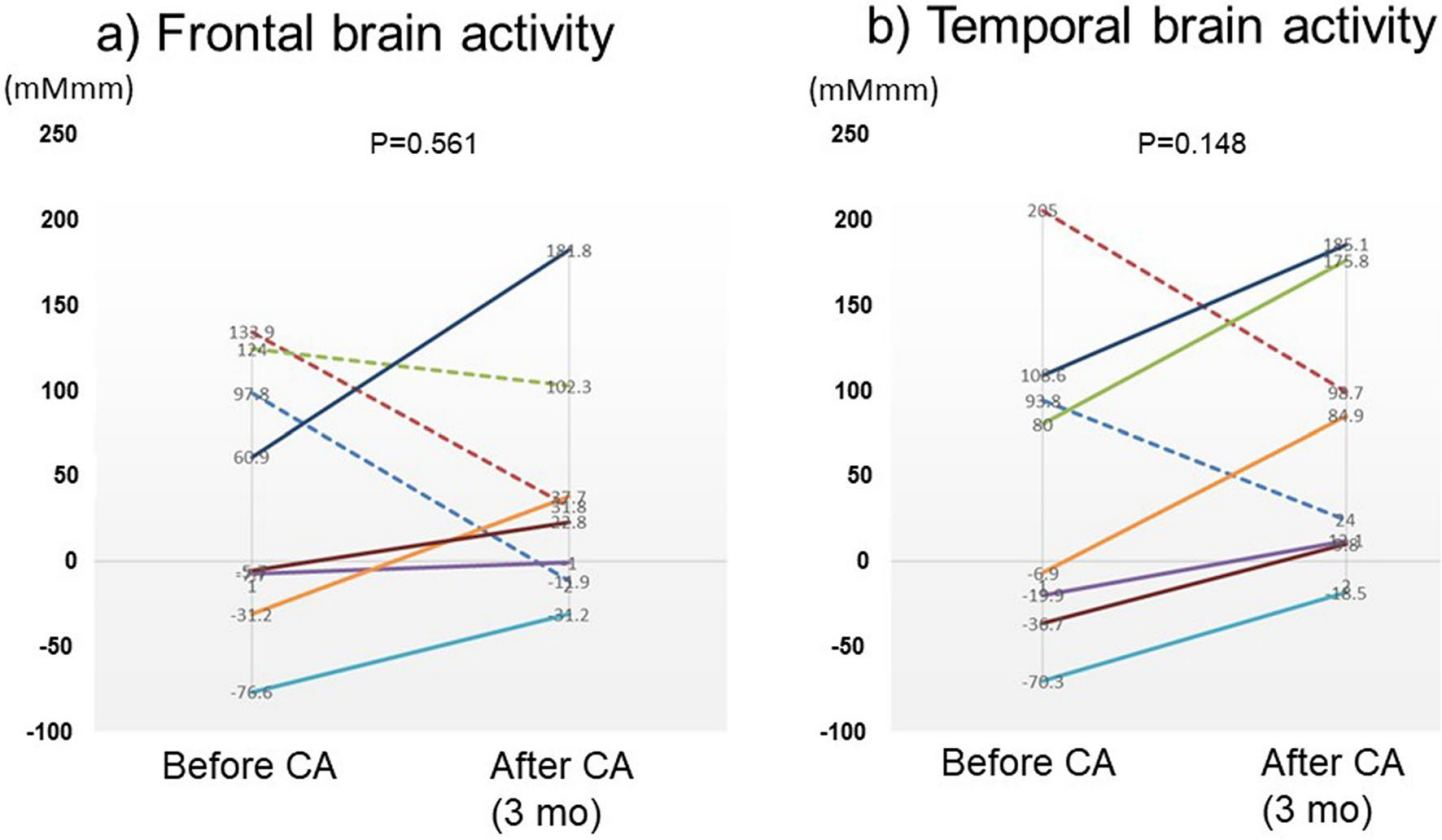
A solid line indicates a patient who showed improvement in brain activity, and a dotted line indicates a patient who showed no improvement. Changes in frontal and temporal brain activities before and after catheter ablation (CA) in the 8 patients with persistent atrial fibrillation (AF) who kept sinus rhythm after CA. Of these 8 patients, 5 (62.5%) presented improved frontal brain activity, and 6 (75.0%) presented improved temporal brain activity after CA.

**Figure 5:**
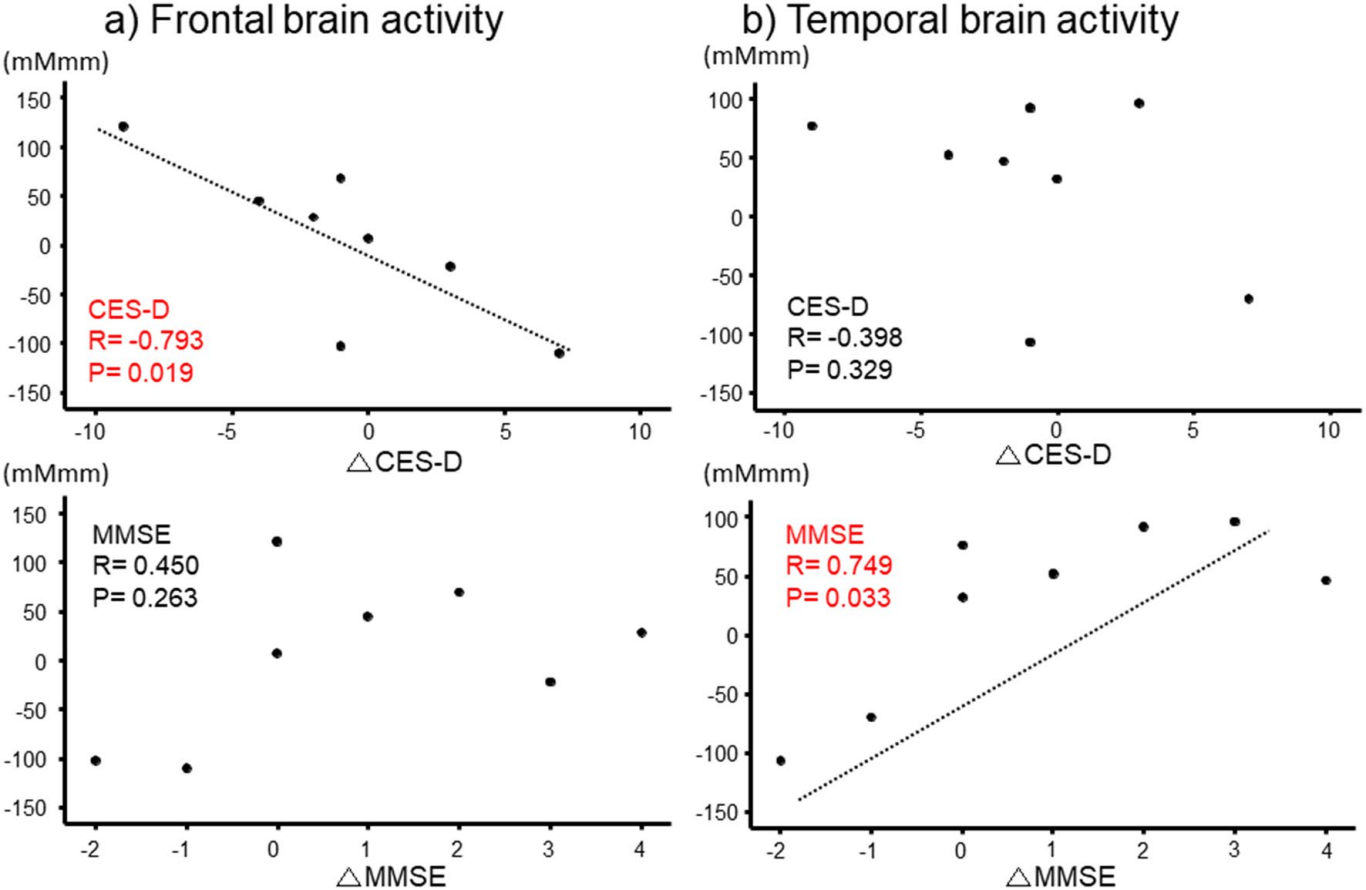
Correlations between changes in brain activities and changes in CES-D and MMSE (n = 8). There were significant correlations of changes in frontal brain activity with changes in CES-D (R =  − 0.793, P = 0.019), but not changes in with MMSE, and there were significant correlations of changes in temporal brain activity with changes in MMSE (R = 0.749, P = 0.033), but not with changes in CES-D.

Of 14 persistent AF patients, 11 patients underwent CA, among whom 8 patients (72.7%) kept SR at 3 months after CA. These 8 patients underwent follow-up examinations of NIRS, CES-D and MMSE 3 months after CA. As shown in Fig. 4, of these 8 patients, 5 patients (solid line, 62.5%) presented improved frontal brain activity, and 6 patients (solid line, 75.0%) presented improved temporal brain activity after CA. Almost of these patients, who presented brain activity as of less than 0 mMmm before CA, seemed to have impaired brain activity before CA and improve brain activity after CA. In addition (Fig. 5), there were significant correlations of changes in frontal brain activity with changes in CES-D (R = − 0.793, P = 0.019), but not changes in with MMSE, and there were significant correlations of changes in temporal brain activity with changes in MMSE (R = 0.749, P = 0.033), but not with changes in CES-D. Thus, (1) frontal and temporal brain activities were improved in some of the persistent AF patients who underwent CA and kept SR, and (2) improvement of frontal brain activity was associated with improvement of depressive state, and improvement of temporal brain activity was associated with improvement of cognitive function.

## Discussion

NIRS has recently been used to investigate not only the neurocognitive processes associated with neurological and psychiatric disorders^20,23,24^, but also neurological hemodynamic changes with cardiac arrest^25^, cardiopulmonary resuscitation^26^, cardiac surgery and cardiovascular anesthesia^27,28^. To the best of our knowledge, the current study is the first to evaluate both brain activity using NIRS, depressive state and cognitive function in AF patients. NIRS showed that (1) frontal and temporal brain activities were lower in the persistent AF patients, (2) frontal and temporal brain activities were improved in some of (over 50%) the persistent AF patients who underwent CA and kept SR at 3 months after CA, especially who presented impaired brain activity before CA, and (3) improvement of frontal brain activity was associated with improvement of depressive state (CES-D), whereas improvement of temporal brain activity was associated with cognitive function (MMSE).

Various AF-related pathologies, including low cardiac output, cerebral hypoperfusion, vascular inflammation, endothelial dysfunction, brain atrophy, thrombosis, genetics factors, and shared risk factors such as hypertension, sleep apnea, diabetes, and obesity, have been shown to affect cognitive function or depressive state^29,30^. Another potential, but unproven, mechanism is brain hypoperfusion following reduced stroke volume and cardiac output attributable to a beat-to-beat variability in the length of the cardiac cycle^13,31^. Moreover, cerebral microbleeds during anticoagulation can also contribute to an impairment of the cognitive function^30^. More severe depressive state was observed in the persistent AF patients than in the paroxysmal AF patients^8^. Reduced cerebral blood flow likely contributes to tissue changes, and thus, has the potential to change depressive symptom levels in AF patients. A decrease in the oxy-Hb concentration measured by NIRS reflects a decrease in frontal brain function in patients with depression or in a depressed state^32^, which may be further associated with cognitive impairment^33^. CA improves clinical symptoms of AF accompanied depressive symptom, can reduce the risk of left atrial thrombosis caused by atrial synchronicity and hemodynamic alternations, and can improve long-term cognitive function^5^. Change in cognitive function before and after the CA of AF, and improvement in the cognitive function 1 year after the CA were reported^5^. Restoring SR after CA improves both the systolic and diastolic function^34^ and may help the recovery from cerebral hypoperfusion and a cognitive impairment in patients with AF. On the contrary, Medi et al.^6^ reported that post-procedural neurocognitive dysfunction partly occurred in AF patients after CA. CA of AF improved neurocognitive function assessed by a brief cognitive screening tool (i.e. The Montreal Cognitive Assessment), especially in AF patients with pre-CA cognitive impairment^5^. This is concordant with the current study where frontal and temporal brain activities were improved in some of (over 50%) the persistent AF patients who kept SR at 3 months after CA, especially in those who presented impaired brain activity before CA.

Study limitations are as follows: First, as a prospective cohort study in a single center with a relatively small number of patients, the present results may not be representative of the general population. Second, since NIRS evaluates only a shallow layer of the brain, deep layers (e.g. hippocampus) or detailed regions could not be evaluated. Although functional MRI is used to accurately evaluate regional cerebral blood flow, high costs and a large-scale device interfere with easy examination. NIRS is superior to MRI for easy-to-repeat measurements. In this study, we evaluated NIRS findings only single measurement in 60 s during VFT. Since NIRS has advantages in high time resolution and easy-to-repeat measurements over MRI, repeated measurement NIRS with short time duration (e.g. every 5 s) may improve the quality of NIRS findings in future study. Third, because of artifacts, some NIRS signals in the temporal lobe could not be fully detected in some study subjects. NIRS signals during the VFT may be influenced by skin blood flow. Fourth, although we excluded the presence of carotid artery stenosis or cerebral infarction before CA, it may have affected cerebral oxy-Hb concentrations due to arteriosclerotic changes. Fifth, general condition may have affected the results of CES-D and MMSE. Sixth, associations between brain activities determined by NIRS and each score of psychological testing (e.g. depression and cognitive function) may not have been precisely examined. Therefore, since the present results should be viewed as preliminary, further prospective studies with a larger population and more frequent measurements of NIRS, CES-D and MMSE are needed.

## Conclusion

NIRS measurement showed reduced frontal and temporal brain activities in the persistent AF patients, CA improved frontal and temporal brain activities in some of these patients, and associated with improvement of depressive state/ cognitive function.

## Methods

### Subjects and study protocol

This is a cross-sectional study with 29 age-matched control subjects and 32 AF patients (paroxysmal AF n = 18 and persistent AF n = 14) who came to Fukushima Medical University Hospital between June 2020 and October 2020. AF was diagnosed and classified as paroxysmal or persistent according to the duration of AF; paroxysmal, ≤ 7 days; persistent, > 7 days^35^. The study subjects underwent echocardiography, carotid artery ultrasonography, laboratory testing, psychological testing and NIRS. The verbal fluency task (VFT), which is commonly used with NIRS analysis, was used to test brain activity^36–38^. The control subjects had no past history of AF, heart failure or structural cardiac abnormalities detected by echocardiography. The study protocol was approved by the ethical committee of Fukushima Medical University (#823), and the investigation conforms to the principles outlined in the Declaration of Helsinki. All subjects provided written informed consent to participate in the study. Patients with carotid artery stenosis, cerebral infarction, dementia, and those receiving treatment for schizophrenia, depression, or bipolar disorder were excluded. Regarding the psychological testing, the Center for Epidemiologic Studies Depression Scale (CES-D) was used to evaluate depressive symptoms^21^, and the Mini-Mental State Examination (MMSE) was used to evaluate cognitive function^22^. We compared the findings of CES-D (depressive symptoms), MMSE (cognitive function) and NIRS (brain activities) between the AF patients and control subjects. Next, we also followed up persistent AF patients who kept SR at 3 months after CA and measured their brain activities using NIRS, CES-D and MMSE after CA to investigate the associations of changes in brain activities with changes in both CES-D and MMSE.

Echocardiography and carotid artery ultrasonography were performed blindly by experienced sonographers using standard techniques^39,40^. The left ventricular ejection fraction was calculated using Simpson’s method in a four-chamber view^39,40^. All measurements were performed using ultrasound systems (ACUSON Sequoia, Siemens Medical Solutions USA, Inc., Mountain View, CA, USA). Blood samples were obtained from all subjects at Fukushima Medical University Hospital. B-type natriuretic peptide levels were measured using a specific immunoradiometric assay (Shionoria BNP kit, Shionogi, Osaka, Japan)^41^.

### Measurement of NIRS

In the current study, oxy-Hb, deoxy-Hb, and total hemoglobin were measured with a 52-channel NIRS machine (Hitachi ETG4000, Hitachi Medical Corp., Tokyo, Japan) using two wavelengths of near-infrared light (695 and 830 nm). Artifacts must be eliminated by having the subject sit in a chair, relax, and move as little as possible. The 52 channels were attached symmetrically around the prefrontal cortex^42,43^. The main measured channels were as follows: right temporal lobe (channels 1–3, 11–14, 22–24, 32–35, and 43–45), left temporal lobe (channels 8–10, 18–21, 29–31, 39–42, and 50–52) and frontal region (channels 25–28, 36–38, and 46–49). Since size of the personal head differed and measurement position in parietal region was easy to be mistaken, we did not use data from channels 4–7 and 15–17^42,43^. An increase in cerebral oxy-Hb concentration in response to the VFT is considered as a marker of brain activity^43^. Most of the lower and forward channels were placed along the line connecting T3-Fpz-T4, based on the international 10–20 system. This system allows prediction of the measurement sites on the brain surface with relatively high accuracy. The sampling rate of oxy-Hb concentration data was 0.1 s. The obtained data were analyzed using the integral mode: the mean oxy-Hb concentrations over the 10-s pre-task period and over the last 5 s of the post-task period were used as the baselines. Oxy-Hb concentrations were measured during a 10-s pre-task period, a 60-s VFT period, and a 55-s post-task baseline period. Linear fitting was applied to the data between these two baselines. The average oxy-Hb concentration during the 60-s VFT period was used for the analysis. A previously reported algorithm^37^ was used to automatically reject data with artifacts. Data are expressed as waveforms and topographic map. The intraclass correlation coefficient of the mean oxy-Hb concentration during the task period was calculated for the 52 channels. The single measure intraclass correlation coefficient was 0.5309, and the average measure intraclass correlation coefficient was 0.6936, which are both reliable, as previously reported^44^. NIRS analyses were performed using MATLAB R2011 (Math Works Inc., Natick, MA, USA), and Prism 6.0 software (GraphPad Software, Inc., San Diego, CA, USA). Changes in hemoglobin oxygenation occur in the subject undergoing the VFT.

### Activation task, VFT

The VFT was used as an activating task during NIRS analysis as previously reported^36–38^. An outline of the VFT procedure is as follows^37,38^. The subject is first prompted by a voice saying, “Start /a/, /i/, /u/, /e/, /o/” to repeat the utterance “/a/, /i/, /u/, /e/, /o/” for 30 s. The baseline activity, which is recorded and is used to remove the effect of vocalization on brain activity from the data. The subject is next prompted by a voice to vocalize as many words as possible that start with a certain letter. This is done in three 20-s sets. The subject is verbally prompted to vocalize words starting with a certain letter to increase the difficulty of the task. The task is scored by recording the number of words uttered in every 20 s. Finally, the subject is prompted by a voice saying, “Stop /a/, /i/, /u/, /e/, /o/” to stop the task and repeat “/a/, /i/, /u/, /e/, /o/” for 70 s.

### Catheter ablation for AF and follow-up

Detailed methods of CA and follow-up have been reported^45–47^. CA was performed for AF following the cessation of all antiarrhythmic drugs for over five half-lives. Circumferential pulmonary vein (PV) isolation was performed using a single-lasso technique while the patient was sedated with dexmedetomidine hydrochloride. A 7-Fr decapolar ring catheter (Lasso, Biosense Webster, Inc., Diamond Bar, CA, USA) and a 7.5-Fr irrigation catheter with a 3.5-mm distal electrode and real time contact force monitoring (ThermoCool SmartTouch SF, Biosense Webster, Inc., Diamond Bar, CA, USA) were inserted into the left atrium via the transseptal approach. After selective PV angiography, radiofrequency energy was delivered with a wide antral ablation line around the PVs, guided by a 3-dimensional mapping system (CARTO system, Biosense Webster, Inc., Diamond Bar, CA, USA). Radiofrequency power output was set at 45–50 W, and contact force was maintained between 10 and 15 g. After circumferential PV isolation, the elimination of the PV potential in each of the upper and lower PVs was confirmed. The endpoint of the PV isolation was defined as the creation of a bidirectional conduction block between the left atrium and PVs. Each patient was followed up at 1, 3, and 6 months after the CA, then every 3 months thereafter. At each visit, 12-lead electrocardiogram and 24-h Holter monitoring were performed. Recurrence was defined as documentation of atrial tachycardia or AF lasting > 30 s recorded in 12-lead ELECTROCARDIOGRAM or 24-h Holter monitoring. Administration of antiarrhythmic drugs after PV isolation depended on the operator’s decision.

### Statistical analysis

Categorical variables are expressed as numbers and percentages. The chi-square test was used for comparisons of categorical variables and followed by Fisher’s exact test when/if appropriate. Normality was confirmed using the Shapiro–Wilk test in each group. Parametric variables are presented as mean ± SD, and non-parametric variables (e.g. BNP, and NIRS findings) are presented as a median and interquartile range. Parametric variables were compared using Student’s t-test, whereas non-parametric variables were compared using the Mann–Whitney U test. To compare continuous variables among the paroxysmal AF, persistent AF and control subjects, Kruskal–Wallis test was used. We performed regression analysis to determine brain activity confounding factors. Correlations of changes in brain activities with changes in CES-D and MMSE before and after CA were assessed using Spearman’s correlation analysis. A P value of < 0.05 was considered statistically significant for all comparisons. All analyses were performed using a statistical software package (SPSS ver. 24.0, IBM, Armonk, NY, USA).

## Data Availability

The data that support the findings of this study are available from the corresponding author upon reasonable request.
